# Cardiometabolic Dysregulation and Heart Failure

**DOI:** 10.31083/RCM38504

**Published:** 2025-05-22

**Authors:** Susannah Ashfield, Utkarsh Ojha

**Affiliations:** ^1^Department of Medicine, Ashford and St Peter’s Hospitals NHS Foundation Trust, KT16 0PZ Surrey, UK; ^2^Department of Cardiology, East Kent Hospitals University NHS Foundation Trust, CT1 3NG Canterbury, UK

**Keywords:** heart failure, metabolic syndrome, cardiometabolic disease, cardiovascular disease

## Abstract

Heart failure (HF) is a complex clinical syndrome resulting from impaired myocardial function or structure, affecting approximately 56 million patients worldwide. Cardiometabolic risk factors, including hypertension, insulin resistance, obesity, and dyslipidemia play a pivotal role in both the pathogenesis and progression of HF. These risk factors frequently coexist as part of cardiometabolic syndrome and contribute to widespread organ and vascular dysfunction, leading to conditions such as coronary artery disease, chronic kidney disease, type 2 diabetes mellitus, non-alcoholic fatty liver disease, and stroke. Emerging evidence suggests that these conditions not only increase the risk of developing HF, but also negatively impact its progression and outcome. As the global burden of cardiometabolic disease continues to rise, a growing number of HF patients will exhibit multiple metabolic comorbidities. Understanding the intricate relationship between cardiometabolic risk factors and diseases and their impact on HF outcomes is therefore crucial for identifying novel therapeutic avenues. A more integrated approach to HF prevention and management—one that considers these interconnected cardiometabolic factors—offers significant potential for improving patient outcomes.

## 1. Introduction 

Heart failure (HF) is a clinical syndrome characterized by cardinal symptoms such as breathlessness, ankle swelling, and fatigue. It arises from a structural and/or functional impairment of the myocardium that results in elevated intracardiac pressure and/or inadequate cardiac output at rest and/or exertion [1]. Recent estimates indicate that the prevalence of HF is approximately 56 million worldwide and projected to increase over the coming decade [2].

A significant contributor in the development of HF is the presence of cardiometabolic risk factors. Hypertension is one of the most prevalent risk factors for HF and can lead to structural and functional changes in the heart, including left ventricular hypertrophy and diastolic dysfunction [2]. Insulin resistance, another prevalent cardiometabolic risk factor, negatively impacts cardiac function via oxidative stress and endothelial dysfunction [3]. Obesity, which is strongly associated with both hypertension and insulin resistance, is an independent risk factor for HF; the increased body mass imposes a greater workload on the myocardium due to elevated cardiac output demands, while also contributing to pulmonary hypertension and right-sided HF [4]. Additionally, altered lipid metabolism has been shown to impact both the development and exacerbation of HF [5].

**Fig. 1.**
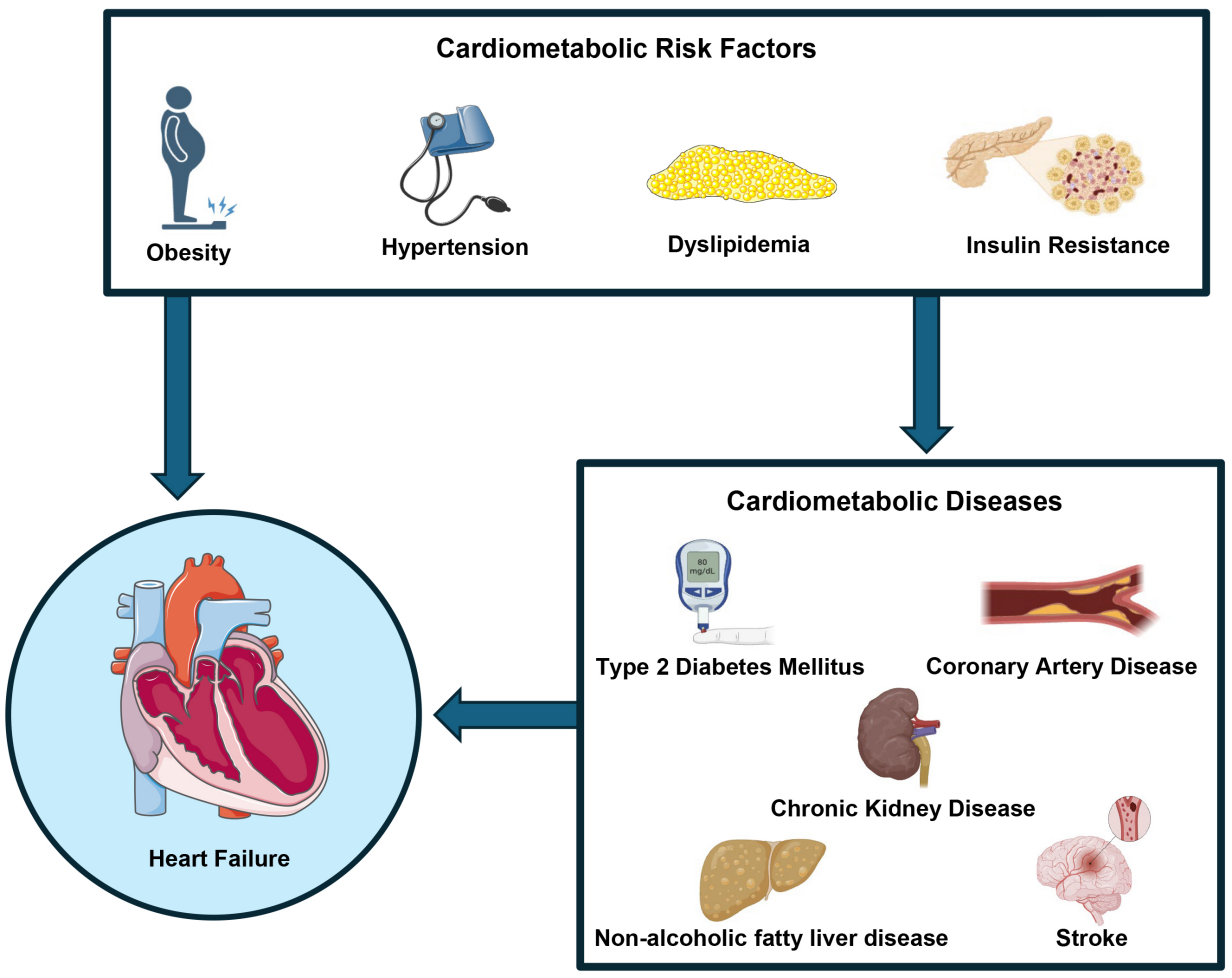
**Cardiometabolic risk factors, including obesity, hypertension, dyslipidemia, and insulin resistance, can contribute to the development of heart failure either directly or through the progression of specific cardiometabolic conditions, such as diabetes mellitus, coronary artery disease, non-alcoholic fatty liver disease, chronic kidney disease, and stroke**. Fig. 1 was created using the image library from https://smart.servier.com/ and https://biorender.com/.

The co-occurrence of these cardiometabolic risk factors is often categorized as cardiometabolic syndrome, which has been associated with a range of cardiometabolic diseases including; coronary artery disease (CAD), chronic kidney disease (CKD), type 2 diabetes mellitus (T2DM), non-alcoholic fatty liver disease (NAFLD) and stroke (Fig. 1). The interlink between cardiometabolic diseases and HF outcomes has become a subject of increasing research interest. It is recognized that CAD increases the risk of HF through reduced coronary perfusion and CKD can worsen HF through mechanisms such as increased blood pressure and inflammation. More recently, stroke and NAFLD have been suggested to impact metabolic dysregulation and cardiovascular dysfunction [6, 7, 8, 9].

As the global prevalence of cardiometabolic diseases continues to rise [10], an increasing number of HF patients are presenting with multiple cardiometabolic comorbidities. Understanding the interplay between these risk factors and diseases and their impact on HF outcomes can open new therapeutic avenues and promote a more integrated approach to HF prevention and management. This review aims to explore the complex relationship between cardiometabolic risk factors, associated cardiometabolic diseases, and HF, by critically evaluating both basic science and clinical trial research to provide a comprehensive overview of the underlying mechanisms. We also discuss current pharmacological therapies supported by evidence for improving HF outcomes, highlight significant gaps in the literature, and propose directions for future research to enhance HF prevention and intervention.

## 2. Cardiometabolic Risk Factors and Heart Failure 

### 2.1 Hypertension 

Hypertension is a leading risk factor for HF, with individuals affected by high blood pressure being 1.5 times more likely to develop HF than those with normal blood pressure [11]. Data from the Framingham Heart Study revealed that hypertension was present in 91% of patients who went on to develop HF. Moreover, the hazard ratio for developing HF in hypertensive patients was nearly double in men and threefold in women, compared to their normotensive counterparts [12].

Cardiac remodelling is the primary mechanism through which hypertensive heart disease develops into HF. A higher arterial pressure generates an increased afterload stress on the heart, forcing the left ventricle (LV) to work harder to eject blood. Over time, this increased afterload leads to concentric left ventricular hypertrophy (LVH), which subsequently reduces the LV chamber volume and impairs diastolic filling, eventually leading to HF with preserved ejection fraction (HFpEF) due to diastolic dysfunction. Additionally, hypertension can cause volume overload when the heart is unable to compensate for sustained high blood pressure. Prolonged volume overload stretches the left ventricle, resulting in left ventricular dilation and HF with reduced ejection fraction (HFrEF). Beyond these hemodynamic changes, hypertension has been shown to induce molecular changes in cardiac myocytes. Cardiomyocyte hypertrophy, apoptosis, myocardial interstitial fibrosis, and alterations in the microvasculature can contribute to the progression of HF [13].

Effectively controlling hypertension helps reverse LVH, prevents acute decompensation episodes, and reduces hospitalizations [14]. In HFrEF, recommended medications include angiotensin-converting enzyme inhibitors (ACE-I), angiotensin receptor blockers (ARBs), beta-blockers, mineralocorticoid receptor antagonists (MRAs), and sacubitril/valsartan. For HFpEF, ACE-I, ARBs, and calcium channel blockers are more effective at reducing LVH than beta-blockers or diuretics [1, 15]. Lifestyle modifications such as weight loss, sodium intake reduction, and increased physical activity are also advised [1]. Current hypertension management in HF emphasizes LVH reversal. However, emerging evidence suggests that targeting myocardial interstitial fibrosis (MIF) may help slow the progression of hypertensive heart disease [16]. Biomarkers like serum procollagen type I carboxy-terminal propeptide (PICP) [17] and imaging techniques such as cardiac magnetic resonance imaging (MRI) are demonstrating promising potential in identifying and assessing MIF [18]. Future approaches may combine these tools for more accurate assessment and targeted therapy, aiming to prevent the transition from hypertensive heart disease to HF.

### 2.2 Obesity 

Obesity contributes towards the development of HF through a variety of interlinked mechanisms. Firstly, obesity leads to an increase in body mass and blood volume which creates greater cardiac workload due to higher cardiac output demands subsequently leading to left ventricular dilatation, hypertrophy and increased LV stiffness [19]. Additionally, a higher density of epicardial adipose tissue intensifies the external constraint exerted by the pericardium on the heart [20]. Furthermore, obesity also leads to changes in cardiac metabolism, resulting in lipotoxicity and reduced efficiency of adenosine triphosphate (ATP) production and utilization [21].

Obesity presents a diagnostic challenge in patients with HF. In obese patients, echocardiogram images may underestimate the degree of congestion [22] and these patients may also have falsely low or normal natriuretic peptide levels [23]. In addition to this, the classical symptoms of HF may be attributed to physical deconditioning due to obesity. Together, these factors can lead to delay in diagnosis and appropriate management of this group of patients.

Although obesity is known to increase the risk of developing HF, the “obesity paradox” suggests that individuals with a moderately elevated body mass index (BMI) experience better HF outcomes compared to those with a lower BMI. A meta-analysis of over 100,000 hospitalized patients revealed that the rate of in-hospital mortality in decompensated HF decreased as BMI increased [24]. Despite this observation, weight reduction in obese patients either by diet, exercise or bariatric surgery has been shown to reverse many of the aforementioned cardiovascular changes that contribute to HF [25, 26, 27]. Indeed, a recent meta-analysis investigating the effect of weight loss in overweight or obese HF patients reaffirmed that weight loss reduced re-hospitalization rates and improved quality of life, cardiac function, and exercise capacity [28]. These contrasting findings may be due to the reliance on BMI for categorizing obesity. Since BMI does not differentiate between lean body mass and adipose tissue, it may not adequately capture the true impact of obesity on HF. Refined risk stratification methods, such as the waist-to-hip ratio, have been suggested to more accurately reflect obesity; studies using this alternative measure have subsequently not supported the obesity paradox [29].

The potential management options for obesity in HF patients range from lifestyle modifications, to pharmacological therapies and surgical intervention. The role of lifestyle modification in HF is the most well-established treatment option; improvements in exercise capacity, as measured by VO_2_, have been reported in patients with HFpEF following caloric restriction and aerobic exercise [30]. Glucagon like peptide-1 (GLP-1) receptor agonists have also emerged as a promising pharmacological option for weight management in HF patients. Kosiborod *et al*. [31] demonstrated that semaglutide, in comparison to placebo, leads to greater weight loss, reduction in symptoms and improved exercise capacity. Recently, Packer and colleagues [32] also reported that in patients with HFpEF and a BMI of greater than 30, treatment with tirzepartide significantly reduced the risk of death from cardiovascular disease and HF. Moreover, bariatric surgery has been shown to result in reversal of LVH with improvements in diastolic function [33]. However, bariatric surgery has significant risks in those with HF; Blumer *et al*. [34] described higher rates of complications following bariatric surgery in patients with HF than those without. Due to inconsistencies in the evidence supporting bariatric surgery for HF, neither the European Society of Cardiology (ESC) nor the American College of Cardiology (ACC)/American Heart Association (AHA) endorse its use [1, 35]. The ACC/AHA guidelines, in particular, emphasize the need for further research in this area [35].

### 2.3 Dyslipidemia

Dyslipidemia is characterized by abnormal levels of lipids in the blood and has been strongly associated with an increased incidence of HF; analysis of the Framingham Heart Study revealed that individuals with higher levels of non-high-density lipoprotein cholesterol (non-HDL-C) and lower levels of high-density lipoprotein cholesterol (HDL-C) experienced higher rates of HF, even after accounting for myocardial infarction (MI) incidence [36].

Dyslipidemia contributes to HF pathogenesis through both direct and indirect mechanisms. Direct myocardial lipid toxicity occurs when excess lipids accumulate within the cardiomyocytes [5, 37]. Under normal conditions, the heart primarily relies on fatty acids for energy. However, in HF, the heart shifts toward increased glucose utilization and decreased free fatty acid metabolism. This metabolic shift results in the accumulation of fatty acids, particularly triglycerides, diacylglycerols, ceramides, and cholesterol, which have been shown to induce cardiomyocyte toxicity. Mouse models confirm that elevated lipid levels in cardiomyocytes result in cardiac dysfunction [38, 39]. Additionally, more oxidized low-density lipoprotein (LDL) cholesterol in humans has been linked to impaired cardiac function [40]. Indirectly, dyslipidemia influences HF outcomes through its association with the development of CAD and T2DM, which are both major risk factors for HF.

Lipid-modifying drugs, particularly statins, have been suggested as a potential therapeutic option to reduce the risk and progression of HF. Statins offer multiple pleiotropic benefits beyond lowering lipid levels, such as promoting LV repair following MI, reducing inflammation, and decreasing oxidative stress [41, 42, 43]. However, two large randomized controlled trials (RCTs) have failed to demonstrate a mortality benefit for statins in HF. The CORONA trial, which examined rosuvastatin in patients with systolic HF, found no reduction in all-cause mortality [44]. Similarly, the GISSI-HF trial, which studied rosuvastatin in patients with New York Heart Association (NYHA) class II-IV HF, showed no reduction in all-cause mortality or cardiovascular hospitalizations [45]. However, concerns have been raised about the generalizability of these trials [46], and subsequent meta-analyses indicated that statin use in HF patients was associated with reductions in all-cause mortality, cardiovascular mortality, and cardiovascular hospitalizations [47, 48]. The evidence for the benefits of statins in HF remains inconclusive. Consequently, current ESC guidelines do not recommend their routine use in HF patients unless there is a separate clinical indication [1].

### 2.4 Insulin Resistance

Insulin resistance contributes to the development of HF by promoting both atheromatous plaque formation and LVH in addition to diastolic dysfunction [49]. These processes occur through a range of molecular mechanisms, including impaired cardiac calcium handling, endothelial dysfunction, reduced cardiac energy efficiency, and changes in substrate metabolism that lead to cardiac lipid accumulation and subsequent lipotoxicity [50, 51].

Several HF medications have been shown to improve insulin resistance [52, 53]. Both ACE-Is and ARBs have been reported to reduce the incidence of new-onset diabetes by 28% and 27%, respectively [53]. Additionally, the cardioselective beta-blocker nebivolol has been shown to improve insulin resistance in murine models [54]. Furthermore, it is suggested that different beta-blockers may have varying effects on insulin resistance. The COMET RCT examined the impact of carvedilol versus metoprolol on the development of new-onset diabetes in HF patients. The results revealed a significantly lower incidence of new-onset diabetes in the group treated with carvedilol [55].

As a potentially reversible cardiometabolic risk factor, insulin resistance presents a key therapeutic target for improving outcomes in HF patients. Lifestyle modifications, such as increased physical activity and reduced caloric intake, have been shown to alleviate tissue insulin resistance [56]. Furthermore, the biguanide metformin has been demonstrated to reverse LV dysfunction in insulin-resistant animal models [57]. The MET-REMODEL RCT investigated the role of metformin in reversing LVH in patients with CAD and pre-diabetes or insulin resistance. After 12 months of metformin treatment, participants showed a significant reduction in LV mass indexed to height, compared to those receiving a placebo [58]. This effect on LVH was proposed to be mediated by a combination of reduction in patients’ systolic blood pressure, reduction in body weight, reduction in oxidative stress, activation of adenosine monophosphate-activated protein kinase (AMPK) and increased insulin sensitivity. However, due to its relatively small sample size (n = 68) and reliance on LV mass as a surrogate marker for cardiovascular outcomes, further RCTs are needed to confirm the role of metformin in HF. Another prospective study into the role of metformin in HF patients showed that patients treated with metformin demonstrated better LV and RV function, lower brain natriuretic peptide (BNP) levels, and better adverse event-free survival [59]. According to current ESC guidelines, metformin is generally considered safe for HF patients with an estimated glomerular filtration rate (eGFR) >30 and no hepatic impairment [1]. However, its efficacy and safety have yet to be rigorously evaluated through RCTs.

### 2.5 Summary 

Certain key cardiometabolic risk factors, hypertension, obesity, dyslipidemia, and insulin resistance, significantly contribute to the development and progression of HF. Hypertension drives cardiac remodeling, leading to both HFpEF and HFrEF, and its management through medication and lifestyle changes can help to reverse LVH. Obesity increases cardiac workload, imposes external constraints on the heart, and alters cardiac metabolism, all of which exacerbate HF. However, weight loss interventions, ranging from lifestyle changes to surgical procedures, can improve outcomes, with further research needed to identify the optimal approach. Dyslipidemia contributes to HF through direct cardiotoxic effects and its role in underlying conditions such as CAD and T2DM. Although current evidence on lipid-modifying treatments in HF is mixed, all studies agree that statins do not cause harm in HF patients. Larger-scale RCTs are needed to better understand the role of statins and identify which patients may benefit from lipid modification. Finally, insulin resistance worsens HF via multiple mechanisms, and metformin shows promise for improving outcomes. Addressing these risk factors with a combination of pharmacological treatments and lifestyle modifications is essential for effective HF management.

## 3. Cardiometabolic Diseases and Heart Failure 

### 3.1 Type 2 Diabetes Mellitus 

The pathogenesis of T2DM is closely linked with the aforementioned risk factors. A higher risk of developing HF has been demonstrated in patients with diabetes as the U.K. Prospective Diabetes Study reported HF incidence rates of 2.3 to 11.9 per 1000 patient-years over 10 years [60]. Additionally, patients with diabetes and HF have been shown to have higher mortality rates than those HF patients without diabetes [61].

**Fig. 2.**
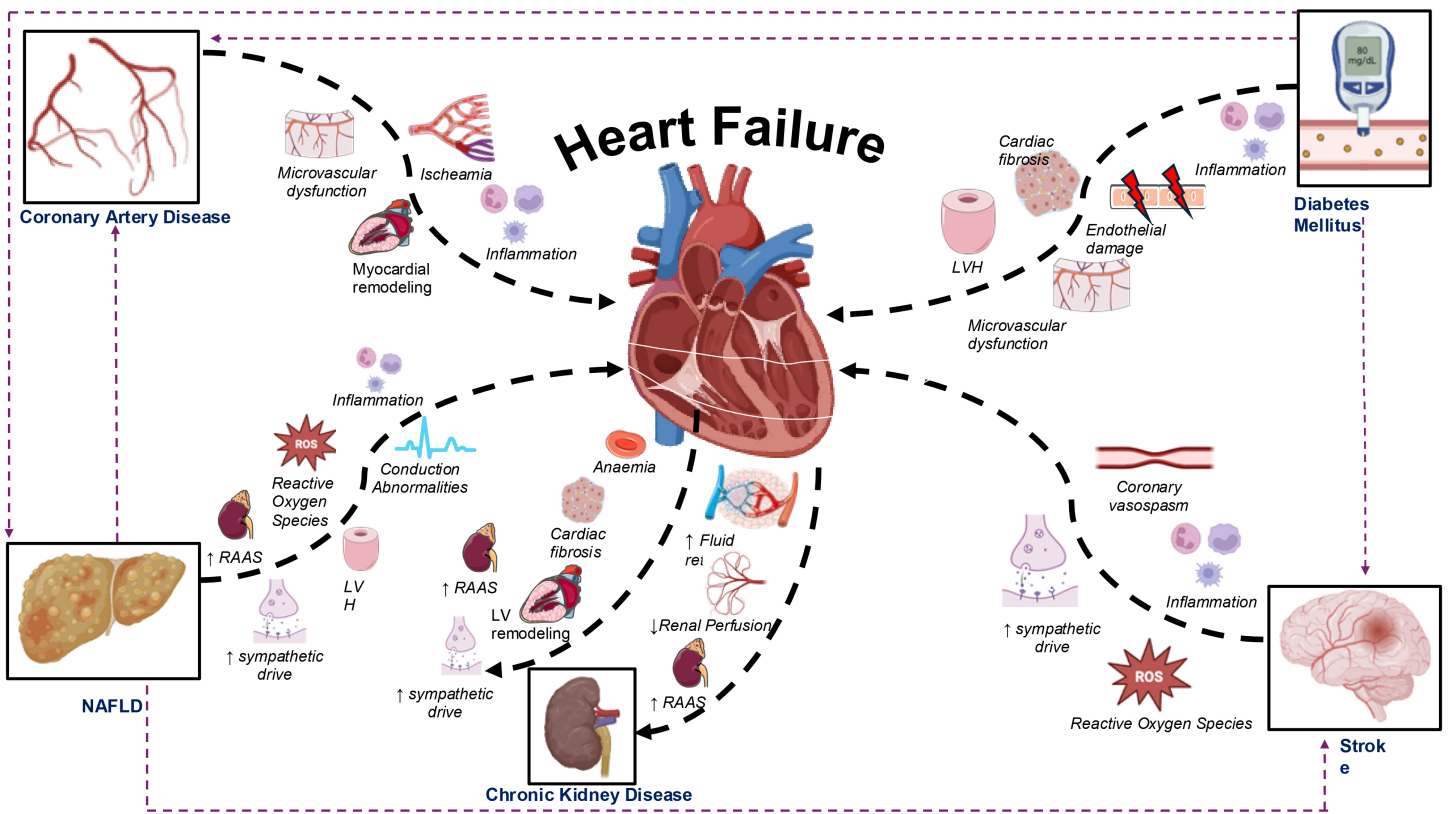
**Mechanisms through which cardiometabolic diseases contribute to the development of heart failure, with particular emphasis on the bi-directional interaction between the heart and kidneys**. NAFLD, non-alcoholic fatty liver disease; LV, left ventricle; LVH, left ventricular hypertrophy; RAAS, renin-angiotensin-aldosterone system; ROS, reactive oxygen species. Thick black dashed arrows represent direct mechanistic influences, while thin purple dashed arrows indicate the development of heart failure through the progression of other cardiometabolic diseases. Fig. 2 was created using the image library from https://smart.servier.com/ and https://biorender.com/.

The effect of T2DM on the heart leads to the development of diabetic cardiomyopathy, which is characterized by LVH, cardiac fibrosis, and diastolic dysfunction. LVH is one of the earliest signs of diabetic cardiomyopathy and higher levels of cardiac fibrosis have been demonstrated in diabetic patients in post-mortem and biopsy samples [62]. T2DM contributes to HF onset and cardiomyopathy through multiple interacting mechanisms including micro- and macrovascular disease, cardiometabolic dysfunction and through alterations in cardiac structure and function. Chronic hyperglycemia and insulin resistance lead to glucose toxicity and lipotoxicity, which damages cardiac myocytes and impairs metabolism. Additionally, diabetes induces oxidative stress and inflammation, leading to further fibrosis and impaired myocardial relaxation. Endothelial dysfunction and microvascular disease reduce blood flow to the myocardium, leading to ischemic damage, which may be further exacerbated by macrovascular coronary artery atherosclerosis. Together, these mechanisms result in structural and functional changes that can lead to both HFrEF and HFpEF (Fig. 2) [63]. Observational studies consistently indicate that poor glycemic control is an independent risk factor for developing HF [64, 65]. Contrastingly, some meta-analytic evidence has suggested that improving diabetic control neither significantly reduces the risk of HF development nor decreases HF decompensation events and may even increase the risk of HF onset [66, 67]. These inconsistencies may stem from variations in the anti-diabetic agents studied, as some have been shown to improve HF outcomes more than others [68].

Sodium-glucose cotransporter 2 (SGLT2) inhibitors are a class of anti-diabetic agents that work by blocking SGLT2 in the proximal convoluted tubule (PCT). This inhibition prevents glucose reabsorption, promoting its excretion through urine [69]. The EMPA-REG OUTCOME trial was the first to demonstrate the beneficial effects of SGLT2 inhibitors in HF, showing a significant reduction in HF hospitalizations among diabetic patients taking an SGLT2 inhibitor [70]. These findings were reaffirmed by subsequent trials including CANVAS and DECLARE-TIMI [71, 72]. The DAPA-HF trial showed a 26% reduction in the incidence of worsening of HF or cardiovascular death in patients with HFrEF taking dapagliflozin irrespective of diabetic status [73]. In the EMPEROR-Reduced trial, patients with HFrEF taking empagliflozin also had a significantly reduced incidence of HF hospitalization and were more likely to improve their NYHA status [74]. Additionally, empagliflozin has shown beneficial effects in patients with HFpEF. The EMPEROR-Preserved trial demonstrated that patients with HFpEF who received empagliflozin had a significantly lower rate of HF hospitalizations compared to those patients who did not receive empagliflozin [75]. Given these consistent findings, the ESC has graded SGLT2 inhibitors as a class I indication for patients with both HFrEF and HFpEF irrespective of diabetic status [1].

The mechanism by which SGLT2 inhibitors exert their cardioprotective effect is multifactorial. They have a variety of effects on the cardiovascular system including: diuretic and antihypertensive effects, promotion of weight loss, increases in hematocrit through erythropoietin production, reversal of adverse cardiac remodeling, improving cardiac energy efficiency by shifting the heart’s metabolism to utilize more ketones and improvements in cardiac calcium handling, which enhances contractility. It has also been suggested that SGLT2 inhibitors may stimulate autophagy, which clears dysfunctional mitochondria and reduces oxidative stress and inflammation in the heart [76].

The impact of GLP-1 agonists on obesity and their potential to improve HF outcomes was explored earlier in this paper. However, until now, no study had specifically evaluated the efficacy of GLP-1 agonist on HF morbidity and mortality. Recently, the SELECT trial examined the effect of semaglutide compared with placebo in high BMI patients with and without a history of HF [77]. Notably, these patients did not have diabetes. The trial specifically evaluated cardiovascular death, hospitalizations, and urgent hospital visits for HF. It showed that treatment with semaglutide reduced composite HF outcomes compared to placebo. A further pooled analysis of four RCTs examined the effects of semaglutide on HF events. The results showed that whilst the effect of semaglutide on cardiovascular death alone was not significant, it significantly reduced the risk of HF and worsening HF events [78]. The benefit of GLP-1 agonists is not only due to improved glucose regulation and weight loss but also through direct cardioprotective actions. GLP-1 receptors are also found in cardiomyocytes, where activation of these receptors promotes myocardial glucose uptake, reduces oxidative stress, and prevents cardiomyocyte apoptosis, offering cardioprotective benefits and preventing adverse cardiac remodeling. Additionally, GLP-1 agonists induce vasodilation by stimulating endothelial nitric oxide production, improving coronary blood flow and reducing blood pressure [79].

Not all antidiabetic agents have been shown to have beneficial or neutral effects in HF patients. Insulin may be harmful due to the increased risk of fluid retention with its use; pooled analysis of RCT data indicates a significantly higher rate of all-cause mortality and HF hospitalizations in patients with HF and diabetes treated with insulin [80]. Sulfonylureas have also been linked to worse outcomes in HF [81]. Additionally, meta-analytic evidence has shown to increase HF decompensation events with thiazolidinediones and therefore these agents are contraindicated in NYHA class III and IV patients [1, 82].

### 3.2 Coronary Artery Disease 

CAD is the leading cause of HF, and its impact is growing, largely due to improved survival rates following acute MI [83]. CAD also serves as a negative prognostic indicator in HF patients and can independently increase HF mortality by up to 250% [84]. CAD can compromise cardiac function both acutely, such as after a myocardial infarction, and chronically over time, resulting in either HFrEF or HFpEF.

The pathogenesis of HF in CAD is multifactorial. Ischemia causes cardiomyocyte death, releasing pro-inflammatory cytokines and attracting leukocytes to the ischemic and surrounding areas, leading to tissue remodeling. This cascade of events extends the area of myocardial damage [85]. Another important factor is coronary microvascular dysfunction, where normal perfusion is not restored following revascularization. The precise mechanisms behind this dysfunction are not fully understood but are thought to involve ischemia-reperfusion injury, intramyocardial hemorrhage, distal embolization, and microvascular obstruction [86, 87]. Moreover, the pathogenesis of HF from CAD can occur through myocardial stunning, hibernation, or remodeling (Fig. 2). Myocardial stunning is a reversible metabolic dysfunction of the myocardium, resulting in a temporary reduction in function after ischemia. In contrast, myocardial hibernation is a chronic, adaptive reduction in myocardial contractility due to prolonged ischemia. In hibernating myocardium, the cardiac muscle reduces its function to conserve energy, maintaining viability despite ongoing blood flow reduction. Compared to stunning, hibernating myocardium suffers greater damage and takes longer to recover after revascularization. At the extreme end of this spectrum, adverse myocardial remodeling occurs, where the myocardium becomes irreversibly scarred and fibrotic [85, 88].

Despite the well-established link between CAD and both the onset and prognosis of HF, CAD evaluation in HF patients remains underutilized [89]. A retrospective review of over 550,000 patients with new-onset HF in the United States found that only 34.8% underwent CAD testing, either invasive or non-invasive [90], highlighting missed opportunities for optimal management in this subset of HF patients.

Once CAD is diagnosed, a myocardial viability assessment is usually undertaken [85]. Some patients may have reversibly stunned or hibernating myocardium, while others may have irreversible scarring. Imaging modalities, such as echocardiography, MRI, single-photon emission computed tomography (SPECT), and positron emission tomography (PET), can be used to assess myocardial viability [85]. Among these, cardiac MRI has emerged as the superior method for determining myocardial viability, demonstrating a sensitivity of 83% and specificity of 88% [91, 92].

The role of revascularization in patients with HF, especially ischemic cardiomyopathy, remains contentious. Previous meta-analyses have demonstrated a survival benefit for patients with ischemic cardiomyopathy and viable myocardium who undergo revascularization compared to those receiving only optimal medical therapy (OMT) [92, 93]. However, a multi-center RCT by Perera *et al*. [94] found that revascularization via percutaneous coronary intervention (PCI) in patients with ischemic LV dysfunction did not significantly reduce all-cause mortality or HF-related hospitalizations compared to OMT alone. The Surgical Treatment for Ischemic Heart Failure (STICH) RCT investigated the effect of coronary-artery bypass grafting (CABG) on all-cause mortality in patients with ischemic cardiomyopathy and an ejection fraction ≤35%. Initial results, with a mean follow-up of 4.7 years, did not show a significant reduction in all-cause mortality. However, the group that underwent surgical revascularization did exhibit lower rates of cardiovascular mortality [95]. The STICH Extension Study, which followed patients for nearly 10 years, demonstrated a significant reduction in all-cause mortality for those undergoing revascularization [96]. A substudy of the STICH trial using myocardial viability testing, however, did not show a survival benefit from surgical revascularization in patients with viable myocardium [97]. This negative finding may have been due to various factors, including the use of multiple imaging techniques, differing thresholds for assessing viability, and the binary definition of viability. Currently, the ESC guidelines recommend surgical revascularization for patients with multivessel CAD and HFrEF (Class Ia indication) with PCI as an alternative option (Class IIb indication) [1]. To date, no RCTs have directly compared the outcomes of PCI and surgical revascularization in HF patients with CAD; the ongoing STICH3-BCIS4 RCT is expected to provide greater clarity on the outcomes of PCI versus CABG in patients with ischemic LV dysfunction and CAD [98].

### 3.3 Chronic Kidney Disease 

HF affects up to 50% of patients with CKD and is among the leading causes of mortality in this population [99]. The heart and kidneys are closely interconnected. The kidneys rely on adequate blood flow from the heart, and cardiac function is influenced by the salt and water balance regulated by the kidneys. This interdependence creates a vicious cycle, where the deterioration of one organ can lead to dysfunction in the other. This interaction can give rise to the development of cardiorenal syndrome [100]. HF exacerbates renal dysfunction by reducing renal blood flow, causing fluid retention, and triggering renal ischemia. Conversely, kidney disease worsens HF through mechanisms such as systemic inflammation, overactivation of the sympathetic nervous system and the renin-angiotensin-aldosterone system (RAAS), development of anemia, uremia and subsequent cardiac fibrosis and remodeling (Fig. 2) [7, 101]. The situation is often further aggravated by common risk factors including; hypertension, hyperglycemia and dyslipidemia, which collectively contribute to the progression of both heart and kidney disease.

Despite this close link between the heart and kidneys, patients with kidney disease have often been excluded from trials studying the management of HF due to concerns about the impaired clearance of the drugs used [102]. Evidence indicates that these patients are less likely to receive guideline-directed medical therapy despite their poorer prognosis [103]. However, substantial research suggests that key medications used in HF management may also offer benefits for kidney disease.

Beta blockers represent one of the four pillars of HF management and concerns exist about their use in patients with renal impairment. However, an analysis of 16,740 individual patients with a left ventricular ejection fraction (LVEF) <50% from 10 double-blind, placebo-controlled trials concluded that there was no significant deterioration in renal function in patients with HF with moderate or moderately-severe renal dysfunction [104]. Indeed, evidence also suggests that beta blockade may have added benefits in patients with CKD. Molnar *et al*. [105] reported an association between beta blockers and reduction in all-cause mortality in elderly patients with CHF and CKD, including those with an eGFR <30. These findings were echoed by Gao and colleagues [106] who studied the use of beta blockers in HF patients on peritoneal dialysis. Although limited due to their retrospective designs, a further meta-analysis of 3136 patients with HF and CKD by Lunney *et al*. [107] described a possible reduction in death with the use of beta blockers relative risk (RR) 0.69, 95% CI 0.60 to 0.79; I^2^ = 0%; moderate certainty evidence).

ACE-Is and ARBs are integral to guideline-directed medical therapy in HF and have demonstrated benefits in CKD, including slowing kidney disease progression and reducing the need for emergency dialysis initiation [108]. However, extensive data on the role of these drugs in patients with both HF and CKD is limited as most HF trials have excluded patients with raised creatinine. The SOLVD trial investigated the role of enalapril in HF and reported a mortality benefit in patients with and without CKD [109]. Conversely, a meta-analysis with over 5000 participants studying the effects of ACE-Is or ARBs in patients with HF and CKD showed that the effect of these drugs on mortality was uncertain [107].

MRAs, such as spironolactone and eplerenone, are also integral in the therapeutic management of HF. Their benefit is also seen in CKD patients; analysis of the RALES data showed that patients with a reduced eGFR and HF showed similar reductions in mortality and hospitalization as those patients with normal renal function [110]. Moreover, the EMPHASIS–HF trial reported that eplerenone reduced mortality in HF patients with CKD. While these patients had a higher incidence of mild hyperkalemia (potassium >5.5 mmol/L), the rates of severe hyperkalemia (potassium >6 mmol/L) were not significantly different from those in the placebo group [111]. This finding supports the safety of eplerenone in CKD when used with careful monitoring.

The beneficial effects of SGLT2 inhibitors for HF patients have previously been discussed. However, due to their renal site of action and diuretic properties, initial concerns were raised about their suitability for patients with CKD. Trials including; DAPA-CKD and EMPA-KIDNEY have demonstrated that these drugs are beneficial in CKD patients by reducing albuminuria, renal decline, and HF morbidity and mortality [112, 113]. Both the DAPA-HF and EMPEROR-Reduced trials included CKD patients and showed that baseline renal function did not reduce the beneficial effect seen on HF morbidity and mortality [73, 74].

Diuretics have a role in providing symptomatic relief by offloading fluid in HF, however they do not improve survival. Their use in patients with CKD is complicated by concerns about worsening renal function, electrolyte imbalances, and diuretic resistance [100]. As renal function declines, diuretics often become less effective due to reduced delivery of the drug to the renal tubules, necessitating larger doses to achieve the same effect [100, 114]. In CKD patients, diuretic resistance can make fluid management more challenging, and close monitoring is essential to avoid hypovolemia, electrolyte disturbances, and worsening renal function. Careful dose titration is required to achieve euvolemia while minimizing any adverse effects.

Early detection of HF in CKD is vital to allow appropriate management and prevention of deterioration. In end-stage CKD, echocardiographic assessment is carried out as part of pre-transplant work up and it is also recommended to regularly assess patients receiving hemodialysis to detect the development of HF [115]. HF prevention strategies should also be implemented in CKD patients. Lifestyle measures, including dietary sodium restriction, exercise prescription and smoking cessation, as well as pharmacological interventions, such as ACE-I use, may decrease the risk of developing HF in CKD patients [116].

### 3.4 Non Alcoholic Fatty Liver Disease 

NAFLD is characterized by the accumulation of fat in the liver in the absence of significant alcohol consumption [117]. It is the most common form of chronic liver disease worldwide, yet cardiovascular disease (CVD) remains the leading cause of death in patients with NAFLD [118]. A systematic review and meta-analysis of over 11 million individuals revealed that NAFLD is associated with a 1.5-fold increased risk of HF, independent of other cardiovascular comorbidities [8]. Notably, this association appears to be stronger in patients with HFpEF than HFrEF [119].

The relationship between NAFLD and HF is multifaceted and involves various pathophysiological mechanisms. These include insulin resistance, overactivation of RAAS and the sympathetic nervous system, increased systemic inflammation and oxidative stress, gut microbiota dysbiosis, as well as genetic and epigenetic alterations (Fig. 2). The liver plays a key role in the development of insulin resistance and elevated insulin levels lead to increased hepatic fat production which further worsens both liver and systemic insulin resistance. This worsening insulin resistance leads to high levels of hyperglycemia, which leads to the production of advanced glycation end products, which may promote cardiac fibrosis. Additionally, inflammation is central to the development of NAFLD, and this systemic inflammation also influences cardiac remodelling through increased production of proinflammatory cytokines like TNF-α and interleukin-6 (IL-6), which impair myocardial function, induce reactive oxygen species (ROS) overproduction, and increase myocardial fibrosis. Excessive free fatty acid production in the liver also leads to ROS overproduction, and these ROS can leak into the systemic circulation and contribute to cardiac remodeling by increasing matrix metalloproteinase activity and impairing calcium handling in cardiomyocytes, leading to diastolic dysfunction. Gut dysbiosis also plays a role in the development of both NAFLD and HF: metabolic stress increases intestinal permeability, allowing bacterial products, such as trimethylamine, to enter the circulation, which promotes both hepatic fat accumulation and cardiac remodeling through ROS production [120]. Collectively, these factors contribute to cardiac electrical remodeling, increasing the risk of arrhythmias, vascular disease, and structural changes such as LVH, which together drive the development of HF [120]. NAFLD has also been linked to propagation of arrhythmias including; atrial fibrillation, QT prolongation, and cardiac conduction block [118, 121]. In a retrospective study of diabetic patients, 31% of those with NAFLD had persistent heart block, compared to only 16% of those without NAFLD [122], suggesting the influence of liver disease on cardiac conduction abnormalities and subsequent HF pathogenesis.

Moreover, NAFLD has been associated with both diastolic and systolic dysfunction, as well as cardiac structural remodeling. A meta-analysis of nearly 34,000 patients found that NAFLD was linked to a lower ejection fraction and worse diastolic function compared to those without the disease [123]. Additionally, in hypertensive patients with diabetes mellitus, NAFLD was found to be associated with LVH [124]. Another critical link between NAFLD and cardiovascular disease is its contribution to atherosclerosis, which can affect the coronary arteries and further promote HF development. A study in Korean men demonstrated that the incidence of coronary and cerebral atherosclerosis was higher in patients with NAFLD, with the risk increasing in parallel with the severity of the liver disease [125].

Both cardiologists and hepatologists must recognize the connection between NAFLD and HF to allow for appropriate risk stratification and identification of patients who may benefit from closer surveillance for the development of either condition. It has been suggested that it is vital for cardiologists to screen HFpEF patients for NAFLD to allow early detection before the development of cirrhosis [126]. Currently, weight loss through lifestyle interventions remains the cornerstone of NAFLD management. However, emerging evidence suggests that medications such as GLP-1 receptor agonists and SGLT2 inhibitors, may provide additional therapeutic options for both NAFLD and HF [127].

### 3.5 Stroke 

Cardiovascular complications are the second leading cause of death after a stroke, and the risk of non-fatal cardiac events, such as HF, also increases post-stroke [9]. Many stroke patients have been found to exhibit reduced LVEF and diastolic dysfunction, although these findings are often limited by a lack of pre-stroke cardiac assessments [128, 129]. Siedler *et al*. [130] investigated 1209 patients with ischemic stroke and observed that 31% showed LV dysfunction, with only one-third of these individuals having been diagnosed with HF before their stroke.

The complex interplay between the brain and cardiovascular system after a stroke can lead to the development of stroke-heart syndrome, primarily due to autonomic dysregulation and systemic inflammation [131]. The brain plays a critical role in regulating heart function through the autonomic nervous system, which modulates heart rate and contractility. Strokes affecting the insular cortex or other brain regions responsible for autonomic control can impair this regulation [131]. Moreover, brain ischemia triggers a surge in catecholamines through increased sympathetic tone. These catecholamines act on β1 and α1 adrenoceptors, generating ROS, leading to coronary vasoconstriction and reduced myocardial blood flow (Fig. 2). This cascade of events causes direct cardiomyocyte toxicity, resulting in LV dysfunction and HF [132]. In some cases, the acute physiological stress of stroke can precipitate Takotsubo cardiomyopathy, a condition in which the left ventricle weakens and balloons apically, impairing its ability to pump blood. Although the exact mechanism of Takotsubo cardiomyopathy remains unclear, the prevailing theory is that it results from a catecholamine surge, like that seen in stroke [133].

Post-stroke inflammation also plays a key role in the development of stroke-heart syndrome [134]. In animal models, stroke-induced cardiac dysfunction is accompanied by systemic inflammation, increased expression of pro-inflammatory cytokines in the myocardium, and infiltration of macrophages into cardiac tissue [135]. Additionally, inflammatory signals from the brain trigger the release of inflammatory cells, such as monocytes and neutrophils, from the bone marrow and spleen [131]. In murine stroke models, a splenectomy reduced the macrophage infiltration, inflammatory cytokine expression and the incidence of post-stroke cardiac dysfunction and cardiac fibrosis [136].

Together, the pro-inflammatory response and autonomic dysregulation following stroke significantly contribute to cardiovascular complications. Physician awareness of the risk of developing HF post stroke is paramount for early detection and treatment. Preventing stroke through management of vascular risk factors, such as smoking cessation, improved diabetic control and lipid management, and lifestyle interventions, such as increasing cardiovascular fitness and nutritional support, can additionally avoid the onset and worsening of HF in this group [137].

### 3.6 Summary

A range of cardiometabolic diseases can contribute to the development and progression of HF. SGLT2 inhibitors have been shown to be a promising agent in patients with diabetes mellitus and HF. However, other antidiabetic agents may be harmful, necessitating careful treatment selection. CAD remains the leading cause of HF, yet it is often underdiagnosed and undertreated. Whilst the evidence suggests a survival benefit from revascularization, particularly with CABG, further research is needed to clarify the role of PCI in this context. CKD accelerates HF progression, yet patients with both conditions often receive suboptimal treatment due to concerns over the renal effects of pharmacological HF therapy, and also the limited inclusion of this subgroup in relevant trials. Additionally, recognizing the link between NAFLD and HF is essential for early risk stratification. Weight loss remains the primary treatment, though emerging therapies such as GLP-1 receptor agonists and SGLT2 inhibitors show promise. Finally, stroke-induced pro-inflammatory and autonomic changes contribute to HF risk, highlighting the importance of stroke prevention in cardiovascular health.

The cardiometabolic risk factors and diseases discussed in this paper are closely interlinked and can exacerbate the development of each other: diabetes mellitus and hypertension are among the leading causes of CKD [138]; obesity and insulin resistance drive NAFLD and T2DM [139]; and dyslipidemia is a risk factor for stroke and CAD [140]. Given their interlinked mechanisms, these cardiometabolic conditions are likely to co-occur in patients, and a multifaceted approach, combining pharmacological, surgical and lifestyle interventions, is essential for optimal management.

## 4. Conclusion

HF remains a poorly understood entity. Emerging evidence has highlighted the pivotal role of cardiometabolic diseases in the pathogenesis and outcome of HF. The interaction between these metabolic disorders can create synergistic effects, exacerbating both the risk and severity of HF. Recent clinical trials targeting these cardiometabolic parameters have shown promise in reducing HF-related mortality. Nevertheless, the global burden of HF is projected to increase exponentially. Future clinical trials should account for the diverse spectrum of cardiometabolic diseases in patients with HF. In particular, there is a need to include CKD patients in clinical trials to allow for a better understanding of the role of HF guideline-directed medical therapy in this group of patients. Further investigation is needed into the role of lipid modification in HF prevention and management given the current conflicting evidence in this area. The role of PCI versus CABG in ischaemic cardiac dysfunction remains unclear, and the outcome of the STICH3-BCIS4 RCT is awaited to provide greater clarity. Additionally, further research is needed to elucidate the precise mechanisms by which NAFLD and stroke impact HF outcomes, potentially uncovering novel therapeutic pathways for targeted intervention. Addressing the growing challenge of HF will require a multifaceted approach, including early identification of at-risk individuals, optimizing cardiometabolic health, and implementing novel and evidence-based therapeutic strategies.
